# The Potassium SK Channel Activator NS309 Protects Against Experimental Traumatic Brain Injury Through Anti-Inflammatory and Immunomodulatory Mechanisms

**DOI:** 10.3389/fphar.2019.01432

**Published:** 2019-11-29

**Authors:** Tao Chen, Jie Zhu, Chun-Hua Hang, Yu-Hai Wang

**Affiliations:** ^1^Department of Neurosurgery, The 904th Hospital of PLA, School of Medicine, Anhui Medical University, Wuxi, China; ^2^Department of Neurosurgery, Drum Tower Hospital, Medical School of Nanjing University, Nanjing, China

**Keywords:** traumatic brain injury, neuroinflammation, SK channels, NS309, TSG-6

## Abstract

Neuroinflammation plays important roles in neuronal cell death and functional deficits after TBI. Small conductance Ca^2+^-activated K^+^ channels (SK) have been shown to be potential therapeutic targets for treatment of neurological disorders, such as stroke and Parkinson’s disease (PD). The aim of the present study was to investigate the role of SK channels in an animal model of TBI induced by controlled cortical impact (CCI). The SK channels activator NS309 at a concentration of 2 mg/kg was administered by intraperitoneal injection, and no obviously organ-related toxicity of NS309 was found in Sprague-Dawley (SD) rats. Treatment with NS309 significantly reduced brain edema after TBI, but had no effect on contusion volume. This protection can be observed even when the administration was delayed by 4 h after injury. NS309 attenuated the TBI-induced deficits in neurological function, which was accompanied by the reduced neuronal apoptosis. The results of immunohistochemistry showed that NS309 decreased the number of neutrophils, lymphocytes, and microglia cells, with no effect on astrocytes. In addition, NS309 markedly decreased the levels of pro-inflammatory cytokines (IL-1β, IL-6 and TNF-α) and chemokines (MCP-1, MIP-2, and RANTES), but increased the levels of anti-inflammatory cytokines (IL-4, IL-10, and TGF-β1) after TBI. The results of RT-PCR and western blot showed that NS309 increased TSG-6 expression and inhibited NF-κB activation. Furthermore, knockdown of TSG-6 using *in vivo* transfection with TSG-6 specific shRNA partially reversed the protective and anti-inflammatory effects of NS309 against TBI. In summary, our results indicate that the SK channel activator NS309 could modulate inflammation-associated immune cells and cytokines *via* regulating the TSG-6/NF-κB pathway after TBI. The present study offers a new sight into the mechanisms responsible for SK channels activation with implications for the treatment of TBI.

## Introduction

Traumatic brain injury (TBI) is defined as damage to the brain resulting from external mechanical force, which is usually caused by vehicle crashes, falls, or physical abuse (Cornelius, 2013). Despite the dramatic improvements in the management of TBI, to date there is no effective pharmacological treatment available to patients, and it remains one of the leading causes of death and disability in children and young adults. The World Health Organization (WHO) predicts that TBI will become the third leading cause of morbidity and mortality by 2020 (Bulstrode, 2014). Neuronal damage after TBI occurs in two phases: the initial primary phase being the injury itself followed by a prolonged secondary damage, which kills a sizable percentage of patients in days to weeks after the event. The delayed phase, including a variety of physiological, cellular, and molecular responses, will result in sustained and progressive neuronal degeneration, but also provide an opportunity in which pharmaceutical compounds with neuroprotective properties could be used (Raghupathi, 2004).

In recent years, increasing evidence has indicated that among various long-lasting pathological changes induced by secondary damage after TBI, neuroinflammation played important roles in neuronal cell death and functional deficits (Corps et al., 2015). TBI induces a strong inflammatory response characterized by glial activation, leukocyte recruitment, and secretion of mediators such as cytokines and chemokines. Posttraumatic cerebral inflammation not only can be detrimental through the release of pro-apoptotic cytokines, reactive oxygen species (ROS), nitric oxide (NO), proteases, and other factors with cytotoxic effects, but also may be beneficial if it is controlled in a regulated manner and for a defined period of time (Correale and Villa, 2004; Ziebell and Morganti-Kossmann, 2010). Thus, finding treatments that aim at minimizing secondary injury by modifying inflammatory response becomes a critical strategy for TBI patients, and some anti-inflammatory agents targeting neuroglia cells activation or inflammatory cytokines have been proved to exert protective effects in both *in vitro* and *in vivo* experiments (Shohami, 1997; Lynch, 2005; Chen, 2011).

Small conductance K^+^ (SK) channels are calcium-activated potassium channels that are present in a wide range of excitable and nonexcitable cells. Four types of SK channels, including SK1, SK2, SK3, and SK4, have been cloned from mammalian systems, and they are demonstrated to be extensively expressed in the nervous system (Drews, 2009; Adelman, 2016). SK channels are activated by a rise in intracellular Ca^2+^, and they are thought to not only contribute to the after-hyperpolarization that follows action potentials, but also play important roles in regulating dendritic excitability, synaptic transmission, and synaptic plasticity (Faber and Sah, 2007). By using pharmacological antagonists or activators, SK channels are shown to be associated with several learning and memory tasks, and also neuroprotective against neuronal injury in neurological disorders, such as stroke (Dolga, 2011; Dolga and Culmsee, 2012; Dolga, 2012–Anderson et al., 2006). More recently, activation of SK channels was shown to exert neuroprotective effects through inhibition of NMDAR-mediated excitotoxicity (Dolga, 2013). In this study, we investigated the therapeutic potential of SK channel activation using NS309 against the TBI-induced neuronal injury, cell death cascades, and neurological dysfunction, and also investigated the potential underlying mechanisms with focus on neuroinflammation.

## Materials and Methods

### Subjects

Male Sprague-Dawley (SD) rats (3 months old, 250–280 g body weight) were purchased from the Animal Experimental Center of Anhui Medical University (totally 216 animals). The animals had continuous access to food and water and were housed in cages in a room maintained at 20°C–22°C with a 12 h light/12 h dark cycle. All experimental protocols and animal handling procedures were performed in accordance with the National Institutes of Health (NIH) guidelines for the use of experimental animals (NIH Publications No. 80-23, revised 1996) and approved by the Ethics Review Committee of Anhui Medical University. All efforts were made to minimize animal number and their suffering.

### TBI Model

TBI was induced by using a controlled cortical impact (CCI) model in according with previously detailed methods (Chen, 2017). Briefly, rats were anaesthetized with an intraperitoneally administered sodium pentobarbital (50 mg/kg) and placed in the stereotaxic frame. A 7-mm-diameter craniotomy was performed over the right cortex midway between the lambda and the bregma. To induce injury, a pneumatic piston impactor device (100 g) with a 4.5 mm diameter and rounded tip was used to impact the brain at a depth of 2 mm (velocity 5 m/s). Then, the scalp wound was closed by standard suture material and the wound area was treated with lidocain cream. During surgery, a warming pad with feedback temperature control ensured a sustained normal body temperature.

### Experimental Design

**Figure 1:**
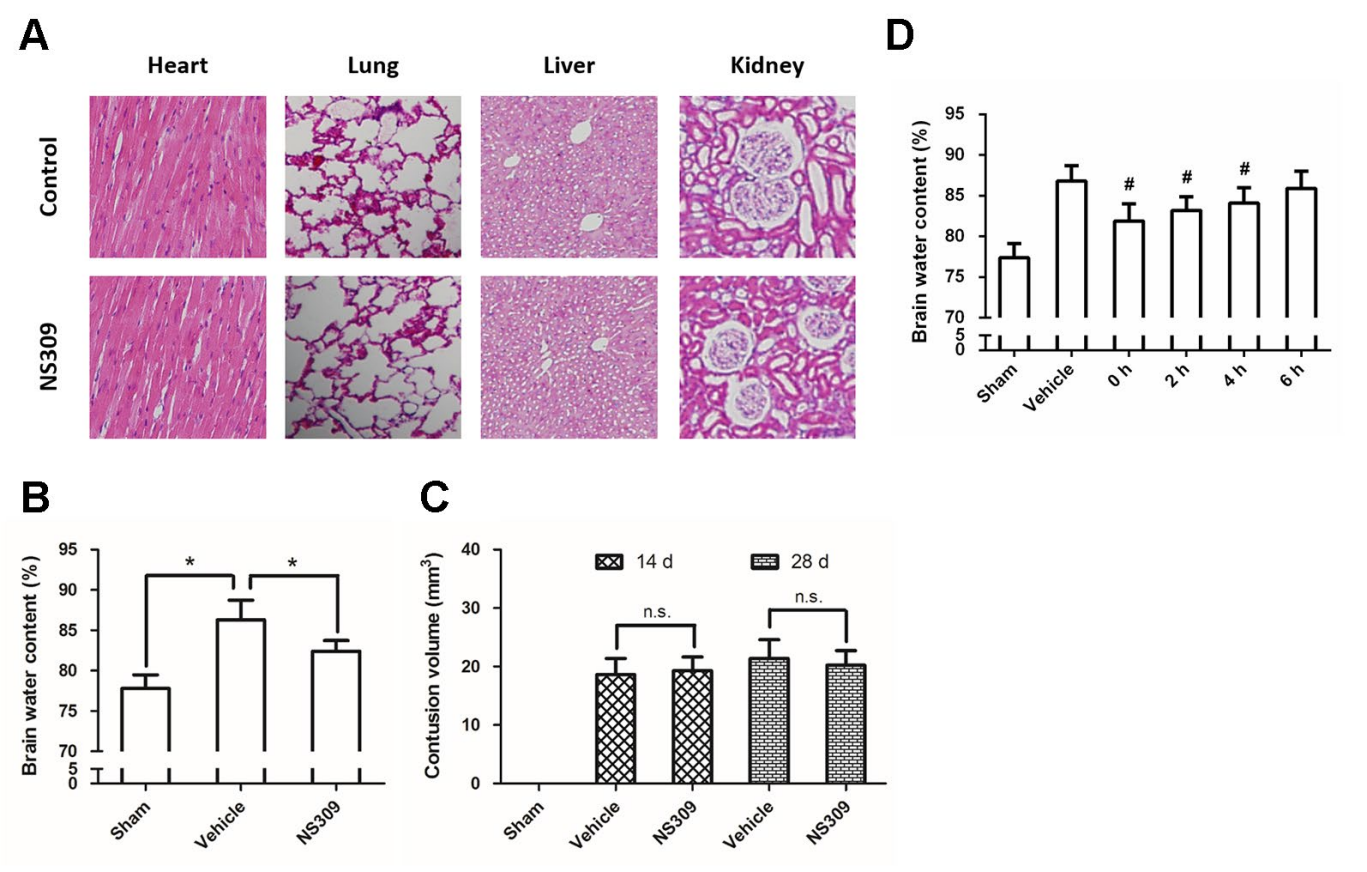
NS309 attenuates brain injury after TBI. The animals were intraperitoneally treated with 0.9% saline (Control) or 2 mg/kg NS309 (NS309) every 2 d for 10 d. After the experiment, animals were sacrificed, and the organs were fixed in formalin overnight and processed for paraffin embedding. The paraffin-embedded blocks were sectioned and stained by hematoxylin and eosin **(A)**. The animals were pretreated with 0.9% saline (Vehicle) or 2 mg/kg NS309 (NS309) 30 min before TBI. Brain edema was measured by brain water content **(B)**, and contusion volume was detected at 14 d and 28 d later **(C)**. The animals were pretreated with 0.9% saline (Vehicle) or 2 mg/kg NS309 (NS309) at different time points after TBI, and brain edema was measured by brain water content **(D)**. Data are shown as mean ± SEM (n = 6). *p < 0.05. n.s., not statistically significant. ^#^*p* < 0.05 vs. Vehicle.

SD rats were randomly divided into three groups (n = 6 each group): sham group, vehicle group and NS309 pretreated group, which was treated with NS309 at a concentration of 2 mg/kg by intraperitoneal injection (100 µl per 20 g) at 30 min before TBI (Dolga, 2011; Zhu, 2019). The animals in sham group were only subjected to surgical procedures, while other animals were subjected to traumatic injury. Saline (0.9%) with 1% DMSO was used as vehicle. Animals were anesthetized and sacrificed at 24 h after TBI for measuring brain water content (**Figures 1B** and **8C**), TUNEL staining (**Figures 3A, B** and **8F, G**), measuring apoptosis factors (**Figures 3C, D** and **8B, H**), and immunohistochemistry (**Figures 4** and **5**); at 14 and 28 d for measuring contusion volume (**Figure 1C**); at 12, 24, and 72 h after TBI for measuring inflammatory cytokines (**Figure 6**); and TSG-6 and NF-κB p65 expression (**Figure 7**).

### Measurement of Brain Edema

Brain edema was determined by measuring brain water content with the wet-dry method at 24 h after TBI. After rats were anesthetized and sacrificed by decapitation, the brains were quickly removed and separated through the inter-hemispheric fistula into left and right hemispheres. Tissue samples from injured hemispheres were weighed immediately to get wet weight. After drying in an oven for 48 h at 100°C, the tissues were reweighed to get the dry weight. Brain water content was then calculated using the following formula: % H_2_O = (1−dry weight/wet weight) × 100%.

### Measurement of Contusion Volume

Contusion volume and brain tissue loss were measured by morphometric image analysis. After rats were anesthetized and sacrificed by decapitation, the brains were quickly removed. At each 500 µm interval, 30 µm sections were mounted on slides and stained with 0.2% cresyl violet solution for contusion volume calculation. The areas of the lesions were integrated, and the results were represented as a volume percentage of the lesion compared with the contralateral hemisphere to avoid the interference from brain edema in the ipsilateral hemisphere.

### Measurement of Neurological Functions

Neurological functions were evaluated using the prehensile traction and beam -balance tests as described previously (Zhao, 2014). The rats underwent pretraining for 3 d prior to randomization. These tests were performed at 1, 2, 3, 7, and 14 d after TBI, and all assessments were performed by an observer blinded to individual treatments. The scoring of prehensile traction test was as follows: 0 = rat grasps the string tightly and climbs up quickly; 1 = rat holds onto the string with its rear limbs and tries to climb beyond 60 s; 2 = rat remains on the string but does not rely on its rear limbs beyond 60 s; 3 = rat falls down from the string during the period of 30–60 s; 4 = rat falls down from the string within 30 s. The scoring of beam-balancing test was as follows: 0 = rat can walk easily and turns around freely; 1 = rat can maintain a stable posture during 60 s; 2 = rat hugs the beam or hooks the wood with its limb beyond 60 s; 3 = rat falls down from the beam during the period of 30–60 s; 4 = rat falls down from the beam within 30 s.

### Morris Water Maze Test

The Morris water maze test was used to evaluate spatial learning and memory function as previously described (Liu, 2013). A circular pool (150 cm diameter, 60 cm deep) was filled with opaque water to a depth of 30 cm and a platform with 12 cm in diameter was placed 1 cm below the water’s surface. All animals were trained for 4 d prior to TBI and then assessed 3 d following TBI. Each animal was given four trials per day, 120 s per trial. For each training trial, a rat was randomly placed in 1 of the 4 quadrants and allowed to swim freely for 120 s or until it found the platform. If the rat was unable to find the platform within 120 s, it was gently guided to the platform by the experimenter and a maximal score of 120 s was assigned. The latency time to reach the hidden platform was recorded by using a computer tracking system. Following the training trials, a probe trial was performed to test spatial memory function. The platform was removed, and the animals were allowed to search freely for 120 s. The number of times each rat crossed the former platform location was measured to assess spatial memory.

### TUNEL Staining

Neuronal apoptosis was measured by TUNEL staining, a method used to observe DNA strand breaks in nuclei. In brief, sections of 4 µm thick were cut, mounted on poly-L-lysine-coated slides, and treated with proteinase K solution (20 µg/ml) for 10 min at room temperature to permeabilize tissues. TUNEL staining was performed by labeling with fluorescein TUNEL reagent mixture for 60 min at 37°C according to the manufacturer’s suggested protocol and examined under a fluorescence microscopy. The number of TUNEL-positive cells in each section in 10 microscopic fields (at ×600 magnification) was counted by an investigator blinded to the grouping.

### Immunohistochemistry

The animals were anesthetized, transcardially perfused, and post-fixed with 4% paraformaldehyde in 0.1 M phosphate buffer at 72 h after TBI. Brain tissues were embedded in paraffin and serial sections were cut at 5 µm intervals from bregma -2.0 mm to bregma -7.0 mm to collect the entire lesioned cortex and mounted on slides. For immunohistochemistry, slides were incubated with primary antibody against glial fibrillary acidic protein (GFAP) (goat polyclonal to GFAP, 1:100, Abcam, New Territories, HK), Ionized calcium binding adaptor molecule 1 (Iba-1) (goat polyclonal to Iba1, 1:100, Abcam), myeloperoxidase (MPO) (rabbit polyclonal to MPO, 1:100, Abcam), and CD3 (rabbit polyclonal to CD3, 1:100, Abcam) at 4°C overnight. Following primary antibody incubation, slides were incubated with secondary antibodies (Alexa 488 donkey-anti-goat IgG, Invitrogen, 1:300; Alexa 594 conjugated goat-anti-rabbit IgG, 1:300) for 2 h, and the negative control was secondary antibody only). All the sections were observed using a fluorescence microscope (Olympus BX51, DP71), and the number of positive cells near the injured areas was counted (8 to 10 sections per brain, 500 µm apart) in a blinded manner.

### Inflammatory Cytokine Analysis

To detect the expression of inflammation-related cytokines, rats were sacrificed at 12, 24, or 72 h after TBI and the brain tissue homogenates were obtained from the injured cerebral hemisphere. The concentrations of IL-1β, IL-6, tumor necrosis factor-α (TNF-α), IL-4, IL-10, transforming growth factor-β1 (TGF-β1), macrophage chemotactic protein-1 (MCP-1), macrophage inflammatory protein-2 (MIP-2), and RANTES were measured using specific ELISA kits according to the manufacturers’ instructions (Boster Biological Technology, Wuhan, China).

### Real-Time RT-PCR

Total RNA was isolated from injured brain tissues at 12, 24, and 72 h after TBI by Trizol (Invitrogen). After equalization of the RNA quantity in each group, levels of TNF-stimulated gene 6 (TSG-6) and nuclear factor-κB (NF-κB) were quantitated using a Bio-Rad iQ5 Gradient Real-Time PCR system (Bio-Rad Laboratories), and GAPDH was used as an endogenous control. Primers for all Real-Time PCR experiments were listed as follow: *TSG-6* primers, 5’-GCAGCTAGAAGCAGCCAGAAAG-3’ (forward), 5’-TTGTAGCAATAGGCGTCCCACC-3’ (reverse); *NF-κB* primers, 5’-CTACACTTAGCCA TCATCCACCTT-3’ (forward), 5’-AGTCCTCCACCACATCTTCCTG-3’ (reverse); *GAPDH* primers, 5’-AAGGTGAAGGTCGGAGTCAA-3’ (forward), 5’-AATGAAGGGGTCATTGATGG -3’ (reverse). The 2^-ΔΔCt^ method was used to calculate the relative expression levels.

### Lentivirus Preparation and Stereotaxic Injection

For developing shRNA lentivirus, a shRNA targeting TSG-6 was purchased from the Santa Cruz Bio-technology (sc-270514-V, TX, USA). The lentiviral construct was co-expressed with green fluorescent protein (GFP) driven by the Ubiquitin C promoter, in addition to U6 promoter 10 driving the shRNA. To control for off-target and non-specific effects of shRNA, a negative control (sc-108066, Santz Cruz, TX, USA) was used. Delivery of lentivirus *in vivo* was carried out by using a stereotaxic cortical injection as previously described (Luo, 2014). Three cortical injections were performed in the right hemisphere (ipsilateral to the lesion) as follows: point 1, 4 mm anterior to the bregma, 6 mm lateral, 6 mm deep; point 2, 0 mm antero-posterior to the bregma, 6 mm lateral, 6 mm deep; point 3, 4 mm posterior to the bregma, 6 mm lateral, 6 mm deep. Each injection contained 3 µl of 1×10^9^ TU/ml lentivirus suspension at a rate of 0.3 µl/min. The needle was withdrawn over a course of 10 min. 5 d after injection of lentivirus rats were subjected to TBI as described above.

### Western Blot Analysis

Homogenates were collected from injured rat brain and the protein concentration was determined using a BCA protein assay kit (Jiancheng Bioengineering Institute, Nanjing, China). Equivalent amounts of protein (40 µg per lane) were loaded and separated by 10% SDS-PAGE gels and transferred to polyvinylidene difluoride (PVDF) membranes. For immunoblotting, membranes were blocked with 5% skimmed milk solution in tris-buffered saline with 0.1% Triton X-100 (TBST) for 1 h, and then incubated overnight at 4°C with the following primary antibody dilutions in TBST: Bax, 1:800; Bcl-2, 1:1200; Cleaved-Caspase-3, 1:600; TSG-6, 1:1000; NF-κB, 1:1000; β-actin 1:1200; GAPDH, 1:1000. After that the membranes were washed and incubated with secondary antibody for 1 h at room temperature. Immunoreactivity was detected by SuperSignal West Pico Chemiluminescent Substrate (Thermo Scientific, IL, USA). An analysis software named Image J (National Institutes of Health, MA, USA) was used to quantify the optical density of each band.

### Statistical Analysis

Statistical evaluation was done with GraphPad Prism software, version 6.0 (GraphPad, San Diego, CA). Significant differences between experiments were assessed by univariate ANOVA (more than two groups) followed by Bonferroni’s multiple comparisons or unpaired *t* test (two groups, one-tailed for western blot and ratio; two-tailed for others). A value of *p* < 0.05 was considered statistically significant.

## Results

### NS309 Attenuates Brain Damage After TBI

To dissect the role of SK channels in TBI, we used NS309, a pharmacological activator of SK channels, and tested its influence on brain damage after TBI. No obviously organ-related toxicity of NS309 was found in our experiments (**Figure 1A**). NS309 at a concentration of 2 mg/kg was administered by intraperitoneal injection (100 µl per 20 g) at 30 min before TBI. Using the wet-dry method, we determined the brain water content at 24 h after TBI (**Figure 1B**). The results showed that TBI caused a significant increase in brain water content, which was partially prevented by NS309 pretreatment. In addition, the contusion volume in injured cerebral hemisphere was also determined at 14 d and 28 d after TBI. As shown in **Figure 1C**, we found that NS309 treatment had no effects on the percentage of contusion volume induced by TBI at both 14 d and 28 d (*P* > 0.05). To determine the therapeutic window of NS309, animals were treated with 2 mg/kg NS309 at different points. Significant reductions of brain water content were observed when NS309 was administrated at 0, 2, or 4 h after TBI, but not when the administration was delayed by 6 h (**Figure 1D**).

### NS309 Preserves Neurological Function After TBI

**Figure 2:**
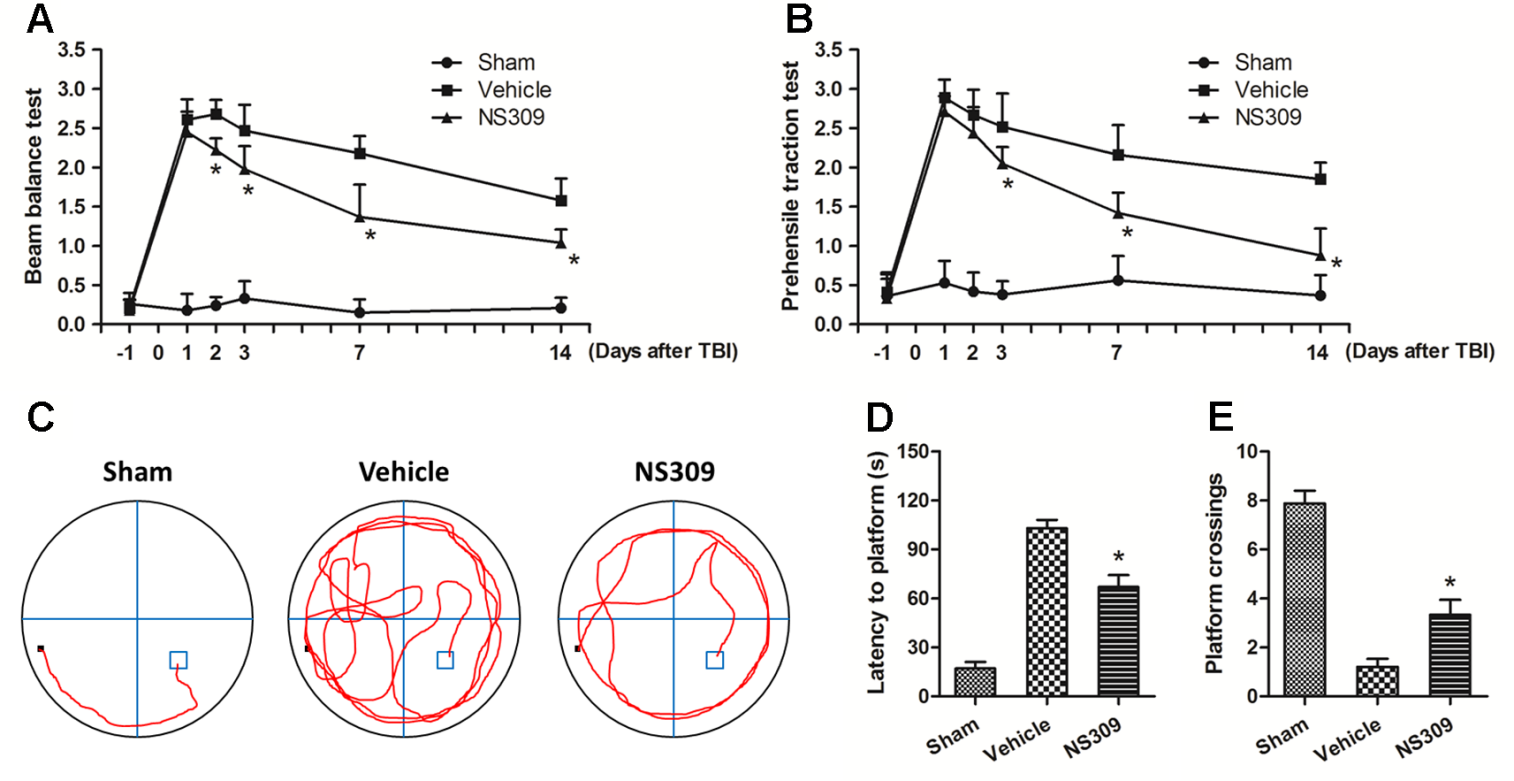
NS309 preserves neurological function after traumatic brain injury (TBI). The animals were pretreated with 0.9% saline (Vehicle) or 2 mg/kg NS309 (NS309) 30 min before TBI. Neurological functions were evaluated using the beam-balance test **(A)** and prehensile traction test **(B)** on days 1, 2, 3, 7, and 14 after TBI. Spatial learning and memory function was evaluated by Morris water maze test **(C)**. The latency time to reach the hidden platform **(D)** and the number of platform location crossings **(E)** were recorded. Data are either representative of three similar experiments or are shown as mean ± SEM (n = 6). *p < 0.05 vs. Vehicle.

To further confirm the neuroprotective effects of NS309, neurological functions after TBI were assayed with the beam balance and prehensile traction tests. Compared with sham group, the neurological scores of both tests were significantly elevated in the vehicle group (**Figures 2A, B**). Pretreatment with NS309 reduced the neurological score of beam balance at 2, 3, 7, and 14 d (**Figure 2A**), and decreased the prehensile traction test score from day 3 to 14 after TBI (**Figure 2B**). In addition, animals pretreated with NS309 or vehicle were also tested in the Morris water maze (**Figure 2C**). As shown in **Figure 2D**, the average latency to the hidden platform in the TBI group was significantly reduced by NS309 pretreatment. In the probe trial, the mean number of platform location crossing was greater in NS309 group than that in vehicle group (**Figure 2E**).

### NS309 Reduces Neuronal Apoptosis After TBI

**Figure 3:**
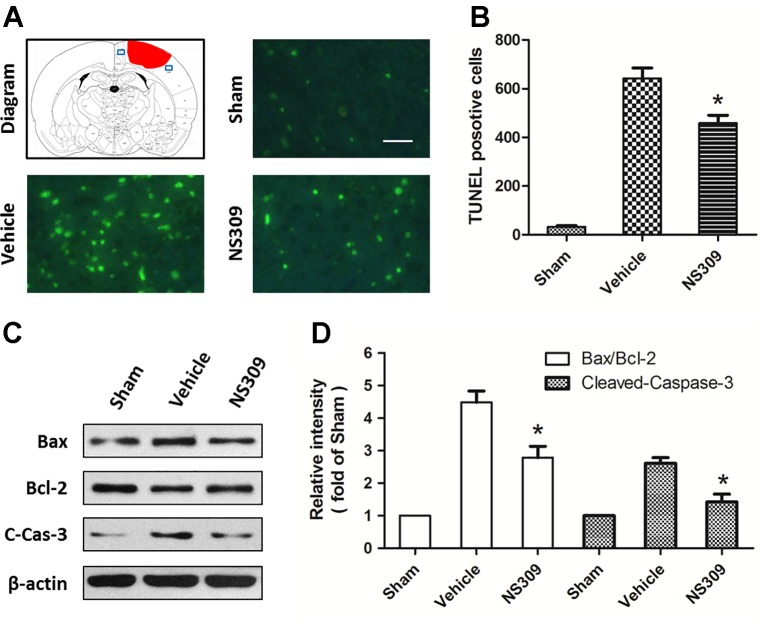
NS309 reduces neuronal apoptosis after traumatic brain injury (TBI). The animals were pretreated with 0.9% saline (Vehicle) or 2 mg/kg NS309 (NS309) 30 min before TBI. Neuronal apoptosis was measured by TUNEL staining **(A** and **B)**, and the expression of Bax, Bcl-2 and cleaved-caspase-3 (C-Cas-3) were detected by western blot **(C** and **D)**. Scale bar: 50 μm. Data are either representative of three similar experiments or are shown as mean ± SEM (n = 6). *p < 0.05 vs. Vehicle.

TUNEL staining was used to detect apoptotic neuronal cell death in the cortex beside the contusion site. As shown in **Figure 3A**, there were few TUNEL-positive apoptotic cells in the cortex of the sham group, and the number of apoptotic cells in the cortex surrounding the injured cortex significantly increased at 48 h after TBI. Treatment with NS309 significantly attenuated the TBI-induced apoptosis as compared to that in vehicle group (**Figure 3B**). To further confirm the anti-apoptotic activity of NS309 treatment, we examined the expression of Bax, Bcl-2, and cleaved caspase-3 by Western blot analysis (**Figure 3C**). The expression of cleaved caspase-3 in the vehicle group was increased as compared with that in sham group, while treatment with NS309 significantly inhibited the activation of caspase-3 (**Figure 3D**). Moreover, NS309 treatment partially reversed the TBI-induced increase in the Bax/Bcl-2 ratio.

### NS309 Inhibits TBI-Induced Activation of Inflammatory Cells

**Figure 4:**
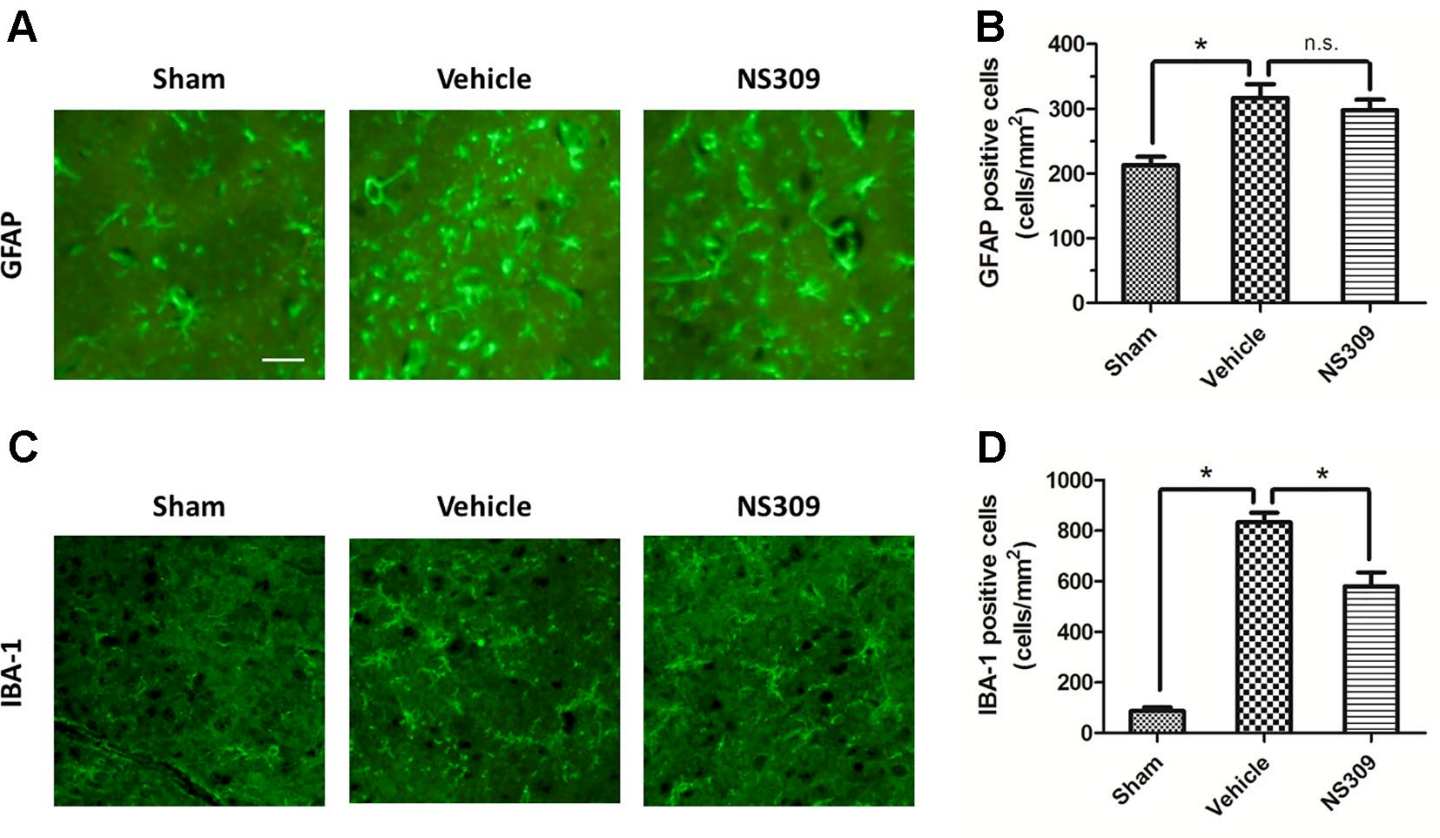
Effects of NS309 treatment on GFAP^+^ astrocytes and IBA-1^+^ microglia. The animals were pretreated with 0.9% saline (Vehicle) or 2 mg/kg NS309 (NS309) 30 min before traumatic brain injury (TBI). Astrocytes were stained with GFAP antibody **(A)** and numbered **(B)**. Microglia were stained with IBA-1 antibody **(C)** and **(D)**. Scale bar: 50 μm. Data are either representative of three similar experiments or are shown as mean ± SEM (n = 6). *p < 0.05. n.s., not statistically significant.

**Figure 5:**
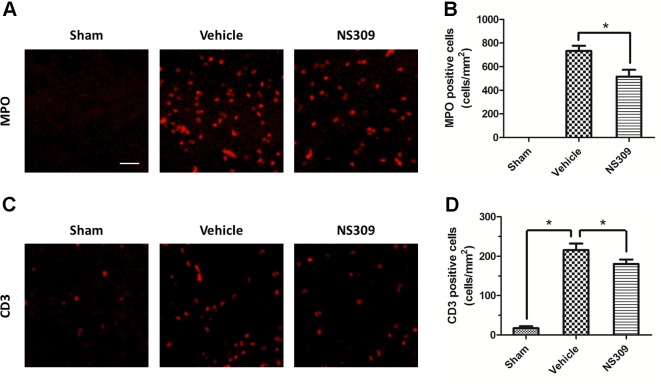
Effects of NS309 treatment on MPO^+^ neutrophils and CD3^+^ lymphocytes. The animals were pretreated with 0.9% saline (Vehicle) or 2 mg/kg NS309 (NS309) 30 min before traumatic brain injury (TBI). Neutrophils were stained with myeloperoxidase (MPO) antibody **(A)** and numbered **(B)**. Lymphocytes were stained with CD3 antibody **(C)** and **(D)**. Scale bar: 50 μm. Data are either representative of three similar experiments or are shown as mean ± SEM (n = 6) *p < 0.05.

To investigate the effects of NS309 on TBI-induced activation of glia cells, we identified GFAP positive astrocytes and Iba-1 positive microglia cells/macrophages in injured cortex by immunohistochemistry (**Figures 4A, C**). TBI increased the number of astrocytes, but the densities of astrocytes were not significantly different between vehicle and NS309 treated group (**Figure 4B**). As shown in **Figure 4D**, the number of microglia/macrophages was significantly reduced in NS309-treated group compared with the vehicle group. In addition, we also detected MPO positive neutrophils and CD3 positive lymphocytes in injured brain to test the effects of NS309 on the number of peripheral infiltrating and resident immune cells (**Figures 5A  C**). The results showed that NS309 significantly decreased the densities of infiltrated neutrophils (Vehicle: 732.4 ± 42.6 cells/mm^2^; NS309: 514.3 ± 58.9 cells/mm^2^) and lymphocytes (Vehicle: 215.6 ± 16.7 cells/mm^2^; NS309: 180.5 ± 11.2 cells/mm^2^) in the injured cortex at 72 h post-TBI (**Figures 5B, D**).

### Effects of NS309 on Cytokine Levels After TBI

**Figure 6:**
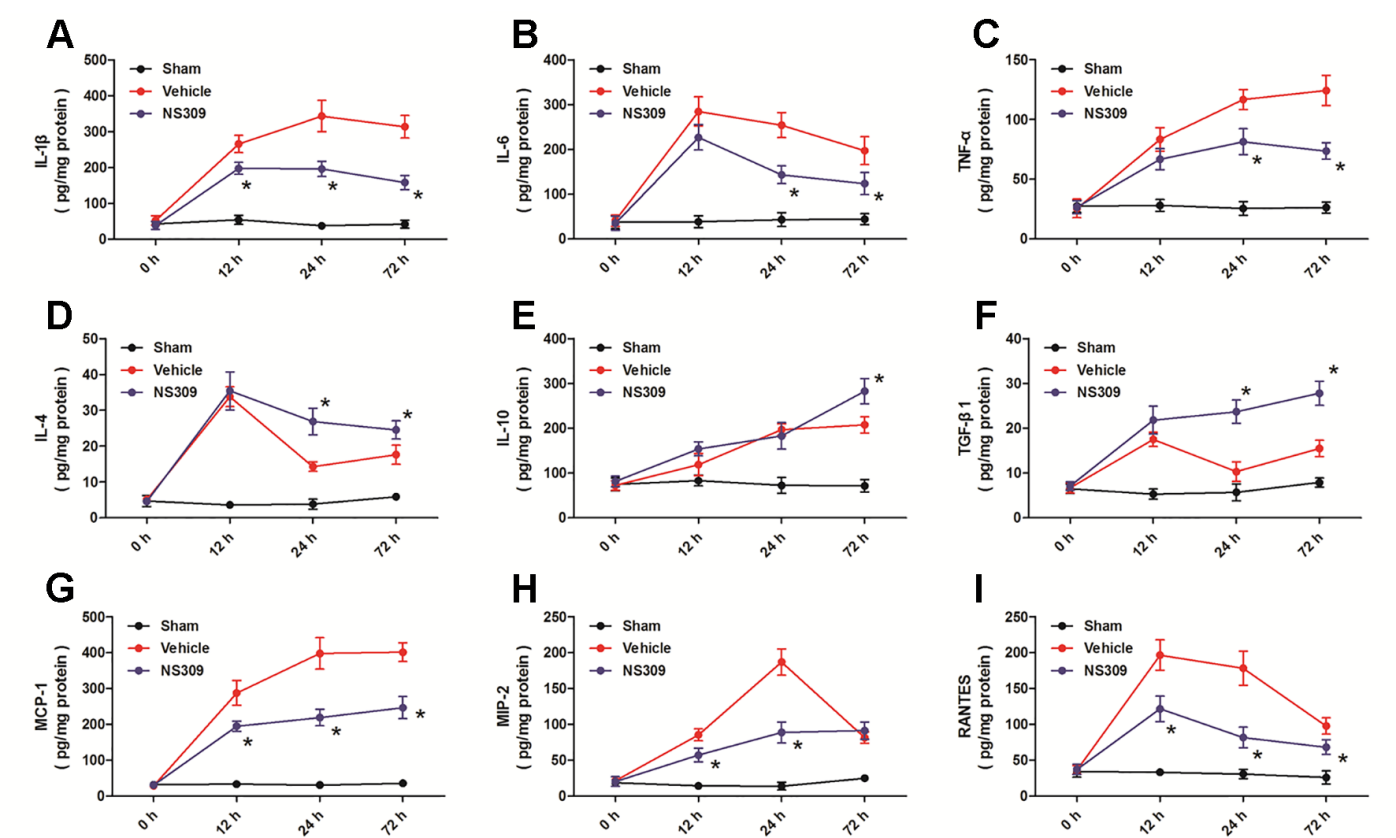
Effects of NS309 on cytokine levels after traumatic brain injury (TBI). The animals were pretreated with 0.9% saline (Vehicle) or 2 mg/kg NS309 (NS309) 30 min before TBI. The expression levels of IL-1β **(A)**, IL-6 **(B)**, TNF-α **(C)**, IF-4 **(D)**, IF-10 **(E)**, TGF-β1 **(F)**, MCP-1 **(G)**, MIP-2 **(H)**, and RANTES **(I)** at 12, 24, and 72 h after TBI were measured, respectively. Data are shown as mean ± SEM (n = 6). *p < 0.05 vs. Vehicle.

To investigate the anti-inflammatory activity of NS309, we assessed an array of inflammatory cytokines in injured cortex homogenates at 12, 24, and 72 h after TBI (**Figure 6**). The expression levels of the pro-inflammatory cytokines IL-1β at 12, 24, and 72 h (**Figure 6A**), IL-6 at 24 and 72 h (**Figure 6B**), TNF-α at 24 and 72 h (**Figure 6C**) after TBI were all significantly reduced by NS309 compared with the vehicle group. In contrast, the expression levels of the anti-inflammatory cytokines IL-4 at 24 and 72 h (**Figure 6D**), IL-10 at 72 h (**Figure 6E**), TGF-β1 at 24 and 72 h (**Figure 6F**) after TBI were all increased in the NS309-treated group compared with the vehicle group. Furthermore, the levels of chemokines MCP-1 (**Figure 6G**), MIP-2 (**Figure 6H**), and RANTES (**Figure 6I**) were all decreased at 12, 24, and 72 h after TBI in the NS309 group compared the vehicle group, although there were no differences in the expression of MIP-2 at 72 h (**Figure 6H**) between the two groups.

### NS309 Regulates the TSG-6/NF-κb Pathway After TBI

**Figure 7:**
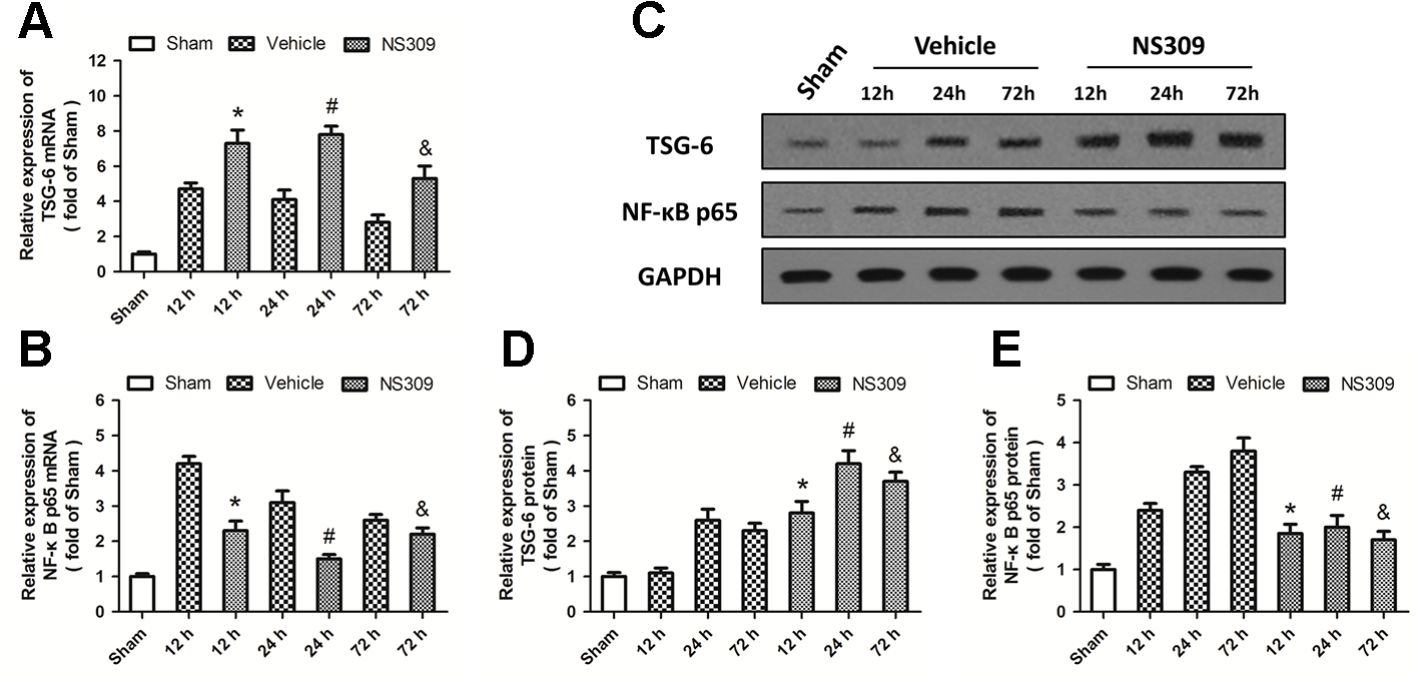
NS309 regulates the TSG-6/NF-κB pathway after traumatic brain injury (TBI). The animals were pretreated with 0.9% saline (Vehicle) or 2 mg/kg NS309 (NS309) 30 min before TBI. The mRNA levels of TSG-6 **(A)** and NF-κB **(B)** were measured by real-time RT-PCR at 12, 24 or 72 h later. The protein levels of TSG-6 and NF-κB from 12 h to 72 h after TBI were detected by western blot **(C)** and calculated **(D** and **E)**. Data are shown as mean ± SEM (n = 6). *p < 0.05 vs. Vehicle at 12 h. ^#^*p* < 0.05 vs. Vehicle at 24 h. ^&^*p* < 0.05 vs. Vehicle at 72 h.

To elucidate the potential molecular mechanisms underlying the NS309-induced anti-inflammatory and immune-modulatory activities, we analyzed the expression of TSG-6 and NF-κB at the mRNA level by real-time RT-PCR. TBI significantly increased the expression of TSG-6 and NF-κB at 12, 24, and 72 h at the mRNA levels. NS309 pretreatment significantly increased the levels of TSG-6 mRNA (**Figure 7A**) but decreased the levels of NF-κB mRNA (**Figure 7B**) at 12, 24, and 72 h after TBI. We also detected the expression of TSG-6 and NF-κB at the protein level by western blot analysis (**Figure 7C**). As shown in **Figure 7D**, the expression of TSG-6 protein was up-regulated by NS309 pretreatment, whereas NS309 significantly inhibited the expression of NF-κB protein at 12, 24, and 72 h after TBI (**Figure 7E**).

### Effects of TSG-6 Knockdown in NS309 Induced Neuroprotection

**Figure 8:**
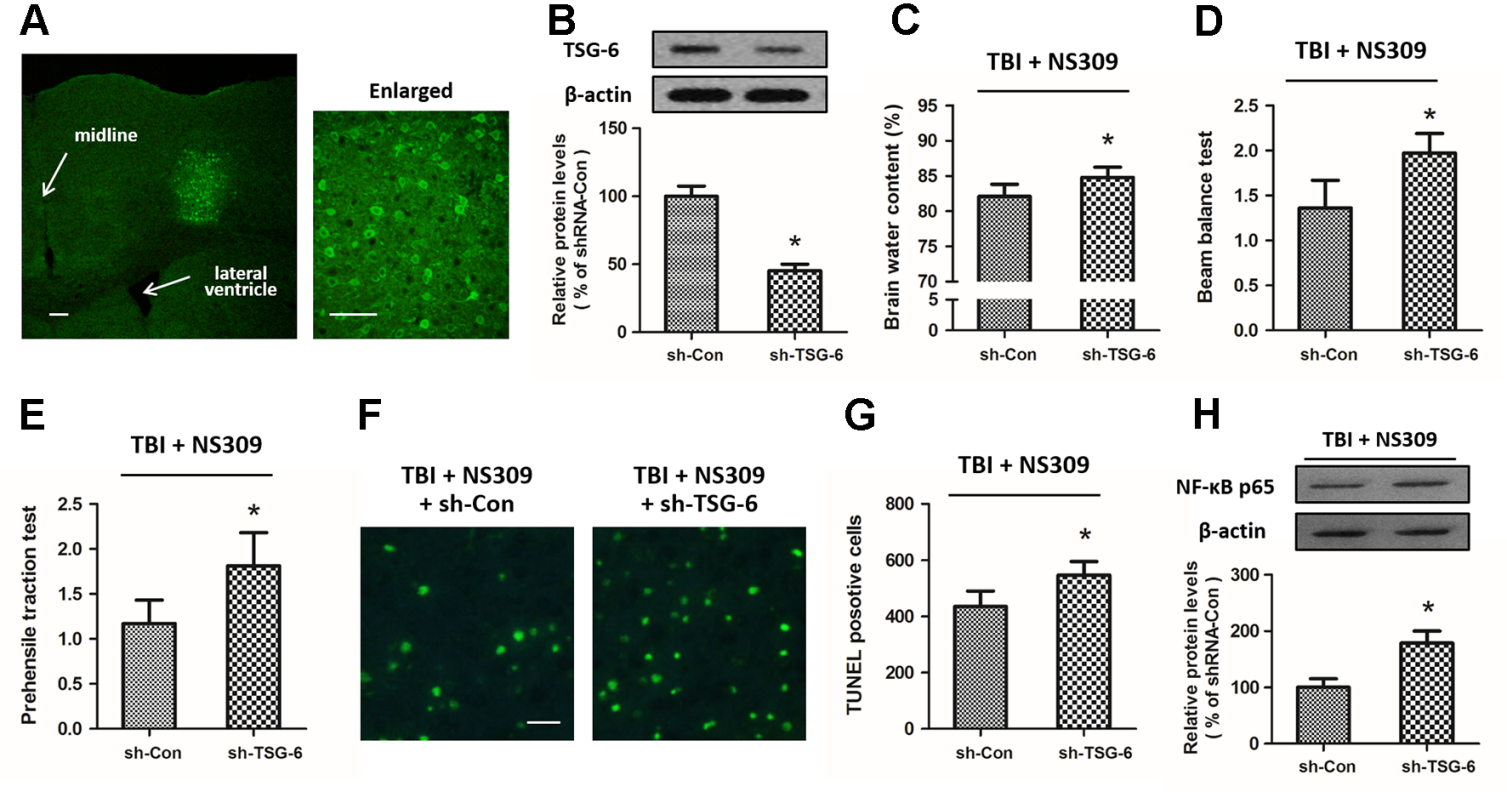
Effects of TSG-6 knockdown in NS309 induced neuroprotection. The animals were injected with lentivirus of shRNA-Control (sh-Con) or shRNA-TSG-6 (sh-TSG-6) in the right hemisphere, and the expression of GFP was detected in an injected area by immunofluorescence staining **(A)**. The expression of TSG-6 protein was examined by western blot **(B)**. After injection with sh-Con or sh-TSG-6, rats were treated with NS309 and TBI was induced 30 min later. Brain water content **(C)** was measured at 24 h after TBI, and neurological functions were evaluated using the beam-balance test **(D)** and prehensile traction test **(E)** at 7 d after TBI. Neuronal apoptosis was measured by TUNEL staining **(F** and **G)**, and the expression of NF-κB was detected by western blot at 24 h later **(H)**. Scale bar: 200 μm in **(A)** left; 50 μm in **(A)** right; 50 μm in **(F)**. Data are shown as mean ± SEM (n = 6). *p < 0.05 vs. sh-Con.

To further investigate the relationship between NS309-induced protection against TBI and its regulatory effect on TSG-6, we infused lentiviruses containing TSG-6 targeted shNRA (sh-TSG-6) or control shRNA (sh-Con) into the right cortex of rats to down-regulate the expression of TSG-6 (**Figure 8A**). 5 d later, western blot analysis revealed that the expression of TSG-6 protein was significantly decreased in the right cortex of the rats (**Figure 8B**). Then, the animals were treated with NS309, and brain trauma was induced in the right hemisphere. The knockdown of TSG-6 partially reversed the protective effects of NS309 against TBI, as demonstrated by the increased brain water content (**Figure 8C**), the increased scores of beam balance test (**Figure 8D**) and prehensile traction test (**Figure 8E**), as well as the increased neuronal apoptosis in the injured cortex (**Figures 8F, G**) in sh-TSG-6 transfected animals compared with sh-Con transfected group. As shown in **Figure 8H**, we also found that the knockdown of TSG-6 partially reversed the inhibitory effects of NS309 on the TBI-induced expression of NF-κB.

## Discussion

Activation of SK channels has been demonstrated to produce neuroprotective effects against glutamate-induced neuronal excitotoxicity, cerebral ischemia, as well as other chronic neurodegenerative disorders (Dolga, 2011; Dolga, 2013). In the present study, we investigated the anti-inflammatory and immunomodulatory properties of SK channel by using its activator NS309 in a rat model of TBI. The main observations were that NS309 significantly attenuated TBI-induced brain edema, neurological dysfunction, and neuronal apoptosis. Pretreatment with NS309 reduced the number of microglia/macrophages in the damaged brain parenchyma and decreased the density of peripheral infiltrating leukocytes in the injured cortex. We also observed decreased expression of pro-inflammatory cytokines and increased anti-inflammatory cytokines levels in injured cortex homogenates after NS309 application. All these effects were partially prevented by knockdown of TSG-6, indicating that the neuroprotective effects of NS309 might be mediated by the increased expression of TSG-6, which in turn suppresses activation of the NF-κB signaling and inhibits the pro-inflammatory cytokine cascades.

Following TBI, increased levels of extracellular glutamate induced by neuronal membrane damage and dysfunction of glutamate transporters lead to an over-stimulation of glutamate receptors, and development of a large cation fluxes, especially calcium influx (Yi and Hazell, 2006). The over-activation of NMDA receptors and followed intracellular calcium overload are demonstrated to be most important factors underlying the enhanced excitotoxicity after TBI and major contributors to the histological damage and neurological dysfunction (Stoica and Faden, 2010). In hippocampal and cortical neurons, SK channels are localized at dendritic spines, where they are closely associated with NMDA receptors, and they can be activated by calcium influx from extracellular space *via* NMDA receptors (Faber et al., 2005; Ngo-Anh, 2005). Activation of SK channels reduces depolarization-induced unblocking of NMDA receptors by external Mg^2+^, thus attenuating Ca^2+^ transient that is crucial to synaptic excitotoxicity and the induction of pro-apoptotic cascades (Ngo-Anh, 2005; Lujan et al., 2009). The Ca^2+^-mediated feedback loop formed by SK channels and NMDA receptors in dendritic spines have been demonstrated, and it might be an important mechanism underlying neuroprotective effects of SK channels in *in vitro* neuronal injury models (Bond et al., 2005). In the present study, we extended the protective effects of the SK channels activator to TBI models, where NMDA receptors mediated excitotoxicity and calcium overload also contribute to the pathology. The results showed that TBI-induced brain edema, neuronal apoptosis, and neurological dysfunction were all attenuated by NS309 pretreatment. SK channels are shown to be expressed in cerebral blood vessels and play a significant role in the regulation of local blood flow, even in ischemic conditions (Marrelli et al., 2003; McNeish et al., 2005). Intriguingly, in the present study, activation of SK channels with NS309 had no effects on TBI-induced cerebral contusion, and these results might be explained by the fact that cerebral infarction after brain ischemia is different from TBI-induced cerebral contusion, which is mainly formed by neuronal necrosis induced by the trauma itself. Otherwise, the stimulation of NS309 on SK channels activities might be limited by the necrotic cell death in the area of cerebral contusion, and these can be revealed by re-expression of functional SK channels in injured cortex after TBI in further researches.

TBI triggers a robust sterile immune reaction that consists of the migration of peripheral inflammatory cells into the brain and the activation of resident cells (Cederberg and Siesjo, 2010). This response is designed to constraint neuronal damage and promote would healing, but it can also become maladaptive if the injury is too severe or too long. Within minutes after TBI, resident microglia are activated to fortify blood-brain barriers and participate in phagocytic cleanup, while neutrophils and monocytes arrive shortly thereafter and preferentially survey injured meningeal spaces (Corps et al., 2015). In the delayed phase post-TBI, there is a complex intercellular interaction between migrating cells accumulated in the brain (such as neutrophils) and resident activated cells (such as microglia), which can aggravate brain damage (Chen, 2011). In our experimental model, immunohistochemistry results showed that the number of GFAP-, Iba-1-, MPO-, and CD3-positive cells were significantly increased after TBI, but all these changes, except for the increased number GFAP-positive cells, were significantly attenuated by NS309, indicating an anti-inflammatory mechanism. The pro-inflammatory cytokines, such as TNF-α and IL-1β, and anti-inflammatory cytokines, including IL-10 and TGF-β1, play opposite roles in inflammation regulation in central nervous system (Spitzbarth et al., 2012). For example, TNF-α mediated tissue damage and functional outcome after TBI (Bermpohl, 2007), and IL-1β induced cortical neuron damage *via* ERK-related mechanism during TBI (Lu, 2005). In contrast, IL-10 and TGF-β1, which have been shown to be upregulated in serum and cerebrospinal fluid in TBI patients, could exert protective effects against TBI through inhibiting free radicals production and astrocytes activation (Fee, 2004; Aisiku, 2016). Here, NS309 treatment was shown to upregulate anti-inflammatory cytokines but downregulate pro-inflammatory cytokines after TBI in rats. Thus, inhibition of inflammatory responses *via* differentially regulating inflammatory cytokines might contribute to NS309-induced protection.

NF-κB is a ubiquitous transcription factor that regulates the expression of many inflammatory genes, including cytokines, chemokines, and adhesion molecules. Inhibition of NF-κB activity has long been recognized as an ideal target for development of anti-inflammatory drugs for many diseases (Lawrence, 2009). Due to the observations that NS309 differently regulated pro-inflammatory, anti-inflammatory cytokines, and chemokines, we speculated that activation of SK channels might inhibit the NF-κB pathway after TBI. The mammalian NF-κB family consists of five subunits, among which p65, also known as RelA, can form dimers with other subunits to mediate downstream gene expression and regulate neuronal survival (Perkins, 2007). The presence of p65 in radiation resistant tissue cells was required for the leukocyte recruitment in the lung after lipopolysaccharide (LPS) treatment (Alcamo, 2001). Thus, we detected the expression of NF-κB p65 in the brain from TBI-injured animals with or without NS309 treatment. The results showed that TBI significantly increased p65 expression, which was partially prevented by NS309 up to 72 h after TBI. Previous studies showed that upregulation of NF-κB could be observed in injured cortex in both TBI patients and experimental TBI animals (Hang, 2005; Hang, 2006). More recently, CD24 was shown to negatively regulate NF-κB pathway to provide a novel target for therapeutic intervention of TBI (Li, 2014). Our results confirmed the expected role of NF-κB in neuroinflammation after TBI and strongly indicated that inhibition of NF-κB activity contributed to NS309-induced protection.

TSG-6, also known as the TNF-induced protein 6 (TNFIP6), is an inflammation associated secreted protein, which is constitutively expressed in many tissues, such as lung, spleen, skin, and brain (Day and Milner, 2018). It was originally cloned from diploid human fibroblasts stimulated by TNF, and high levels of TSG-6 protein have been found in synovial fluids of patients with inflammatory joint disorders, as well as in the sera from bacterial sepsis and systemic lupus erythematosus patients (Day and Milner, 2019). In this study, increased levels of TSG-6 mRNA and protein were observed at 12, 24, and 72 h after TBI. TSG-6 is induced by pro-inflammatory cytokines, including TNF and IL-1, but conversely, it can inhibit inflammation through attenuating neutrophil infiltration, making it a component of a negative feedback loop of inflammation response (Wisniewski, 1996). Previous studies showed that the anti-inflammatory mechanisms of TSG-6 were related to inhibition of the protease network, downregulation of plasmin activity, suppression of neutrophil extravasation, as well as promotion of inflammation resolution *via* cyclo-oxygenase-2 (COX-2) and prostaglandin D_2_ (PGD_2_) (Cao, 2004; Mahoney, 2005; Mindrescu, 2005). More importantly, TSG was shown to suppress NF-κB signaling in resident macrophages (Choi, 2011). Our results showed that induction of TSG-6 protein was required for the NS309-induced NF-κB inhibition and neuroprotection against TBI. The beneficial effects of TSG-6 treatment has been demonstrated in several disease models, such as myocardial infarction, wound healing, acute lung injury, and type-1 diabetes (Lee, 2009; Mittal, 2016; Liu, 2016; Kota, 2013). Watanabe et al. showed that intravenous administration of TSG-6 preserved blood brain barrier and improved memory function after TBI (Watanabe, 2013). In addition, TSG-6 has been shown to be expressed in GFAP-positive astrocytes and participate in astrocyte maturation (Coulson-Thomas, 2016). This might partly explain why NS309 treatment increased TSG-6 expression with no effect on TBI-induce astrocyte activation.

There are some limitations to our study. First, NS309 was discovered as a modulator that activates both SK and intermediate calcium-activated potassium (IK) channels (Strobaek, 2004). IK channel is another type of calcium-activated potassium channels, and it seems not to be present in central neurons, but is expressed in blood, glia, and epithelial cells (Pedarzani and Stocker, 2008). Previous studies have shown that blocking the activation of IK channels could reduce inflammation after traumatic and ischemic brain injury when the agents was applied as late as 12 h after the injury (Pedarzani and Stocker, 2008). Thus, whether some IK channels-mediated mechanisms were also involved in the results observed in this study needs to be further determined in the future. In addition, our results about the changes of NF-κB expression were obtained from the whole brain extracts. It is well known that the regulatory effects of the NF-κB pathway in CNS are associated with neurons, glia cells, as well as immune cells (Albensi, 2001; Aloor, 2015). Some more experiments analyzing the expression of these factors in different cell types separately might be helpful for confirming our conclusion.

In summary, our present study demonstrated for the first time that the SK channels activator NS309 protects against experimental TBI and that these protective effects are partially dependent on the TSG-6/NF-κB pathway-mediated regulation of neuroinflammation. These data help determine the role of SK channels in TBI and indicate a therapeutic potential of NS309 for TBI.

## Data Availability Statement

The mRNA data can be found in GenBank using the accession number 5265937.

## Ethics Statement

All experimental protocols and animal handling procedures were performed in accordance with the National Institutes of Health (NIH) guidelines for the use of experimental animals (NIH Publications No. 80-23, revised 1996) and approved by the Ethics Review Committee of Anhui Medical University.

## Author Contributions

TC and Y-HW conceived and designed the experiments. TC and JZ performed the experiments. C-HH analyzed the data. TC wrote the paper.

## Funding

This work was supported by the National Natural Science Foundation of China (No. 81701932, No. 81871589, and No. 81601719), the Clinical Medical Science and Technology Development Foundation of Jiangsu University (No. JLY20180028), and the China Postdoctoral Science Foundation funded project (No. 2019M651803).

## Conflict of Interest

The authors declare that the research was conducted in the absence of any commercial or financial relationships that could be construed as a potential conflict of interest.
